# Association Between Unenhanced CT Features and Perioperative Outcomes in Bronchogenic Cyst Resection

**DOI:** 10.3390/diagnostics16183000

**Published:** 2026-09-16

**Authors:** Kai-Man U, Pei-Hsing Chen, Min-Shu Hsieh, Yu-Sen Huang, Tzu-Pin Lu, Hsao-Hsun Hsu, Jin-Shing Chen

**Affiliations:** 1 Division of Thoracic Surgery, Department of Surgery, National Taiwan University Hospital, Taipei 100225, Taiwanchenph@ntu.edu.tw (P.-H.C.);; 2 Division of Thoracic Surgery, Department of Surgery, Fu Jen Catholic University Hospital, New Taipei City 24352, Taiwan; 3 Institute of Biomedical Engineering, National Taiwan University, Taipei 10617, Taiwan; 4 Department of Pathology, National Taiwan University Hospital, Taipei 100225, Taiwan; 5 Graduate Institute of Pathology, National Taiwan University College of Medicine, Taipei 10051, Taiwan; 6 Department of Pathology, National Taiwan University Cancer Center, Taipei 106037, Taiwan; 7 Department of Medical Imaging, National Taiwan University Hospital, Taipei 100225, Taiwan; 8 Department of Radiology, National Taiwan University College of Medicine, Taipei100225, Taiwan; 9 Institute of Epidemiology and Preventive Medicine, College of Public Health, National Taiwan University, Taipei 100225, Taiwan; 10 Department of Surgical Oncology, National Taiwan University Cancer Center, Taipei 106037, Taiwan

**Keywords:** bronchogenic cyst, computed tomography, postoperative outcome

## Abstract

**Background**: Reliable predictors of perioperative risk beyond symptomatology in bronchogenic cyst (BC) resection are lacking. We aimed to compare perioperative outcomes across different imaging features to identify perioperative risk factors. **Methods**: We conducted a retrospective cohort study of 57 patients who underwent pathologically confirmed BC resection at a single tertiary center from 2004 to 2023. Preoperative unenhanced CT images were retrospectively reviewed for four prespecified imaging features. Only unenhanced images were used for imaging-feature classification and Hounsfield-unit measurement. Patients were stratified into two groups based on the presence (*n* = 19) or absence (*n* = 38) of abnormal features. The prespecified primary perioperative outcomes were peri-cystic adhesion, operative time, estimated intraoperative blood loss, and intensive care unit (ICU) length of stay. Secondary outcomes included chest-tube duration, postoperative hospital length of stay, major intraoperative complications, 30-day postoperative complications, and recurrence. **Results**: The abnormal image-feature group had a higher prevalence of male patients, symptomatic presentation, and larger cysts. Nineteen patients (33%) had at least one abnormal CT feature. Patients with abnormal CT features had significantly higher peri-cystic adhesion rates (68% vs. 34%; *p* = 0.015), longer mean operative times (128 ± 60 vs. 87 ± 35 min; *p* = 0.011), greater blood loss (100 ± 150 vs. 4 ± 24 mL; *p* = 0.012), and prolonged intensive care unit stays (0.84 ± 1.21 vs. 0.05 ± 0.23 days; *p* = 0.011) than those with normal imaging. No significant differences were observed in outcomes of chest-tube duration, postoperative hospital length of stay, or recurrence. The presence of abnormal features and cyst size remained significant in multivariate models. **Conclusions**: Abnormal imaging features on non-contrast CT and cyst size are associated with more challenging perioperative courses in BC resection. Incorporating these imaging features into preoperative assessment may enhance risk stratification and guide surgical approach decisions in the CT screening era.

## 1. Introduction

Bronchogenic cysts (BCs) are rare congenital malformations originating from the endoderm during respiratory system development. With the global implementation of lung cancer screening programs [1,2], the prevalence of BCs in the general population may be on the rise [3,4].

Patients with mediastinal BCs may remain asymptomatic, and cyst enlargement can lead to symptoms caused by compression of adjacent organs, infection, hemoptysis, and, in rare cases, malignant transformation. Therefore, surgical resection is recommended for all suspected BCs in operable candidates [5,6]. Reportedly, surgical resection has a morbidity rate of up to 27%, posing a significant challenge when treating BCs [5,6]. Early surgical removal of BCs enhances pulmonary parenchyma preservation, decreases the incidence of inflammatory lesions, and lowers the overall complication rate. Recent advancements in video-assisted thoracoscopic surgery (VATS) further support early intervention [7,8,9,10], because this procedure is a safe and effective approach for treating BCs [11].

However, no reliable predictive factor has been established for intraoperative and postoperative morbidity, with symptoms being the only indicators of intraoperative complications [12]. Advancements in imaging techniques provide more detailed image characterization of BCs and their correlation with histopathologic features, thus enhancing our ability to reassess morbidity risk more accurately in the era of computed tomography (CT) screening [13]. We therefore hypothesized that four predefined CT features—lobulation, calcification, an air–fluid level, and heterogeneous attenuation—would be associated with greater perioperative complexity. The prespecified primary perioperative outcomes were peri-cystic adhesion, operative time, estimated intraoperative blood loss, and ICU length of stay. Secondary outcomes included chest-tube duration, postoperative hospital length of stay, major intraoperative complications, 30-day postoperative complications, and recurrence. Therefore, we aimed to compare perioperative outcomes across different imaging features to identify potential risk factors.

## 2. Materials and Methods

### 2.1. Data Collection

We retrospectively reviewed patients diagnosed with BCs, confirmed through pathological examination after surgical excision, at the National Taiwan University Hospital between January 2004 and December 2023. The sample size was determined using the total eligible patient population over the study period. All consecutive patients with pathologically confirmed bronchogenic cysts during the inclusion period were identified from the institutional pathology database and screened for eligibility. Source data were cross-checked against electronic medical records, operative notes, pathology reports, and the institutional imaging archive. Curative-intent surgery was defined as an operation planned to achieve complete cyst excision without intentional gross residual disease. Procedures in which cyst tissue was obtained incidentally during surgery for another condition and procedures performed by non-thoracic surgical teams were excluded. No patient otherwise meeting the imaging eligibility criteria had incomplete clinical or perioperative documentation for the analyzed variables. If repeat procedures were performed for the same lesion, only the first curative-intent resection was included as the index operation, and subsequent procedures were recorded as recurrence-related events.

**Figure 1:**
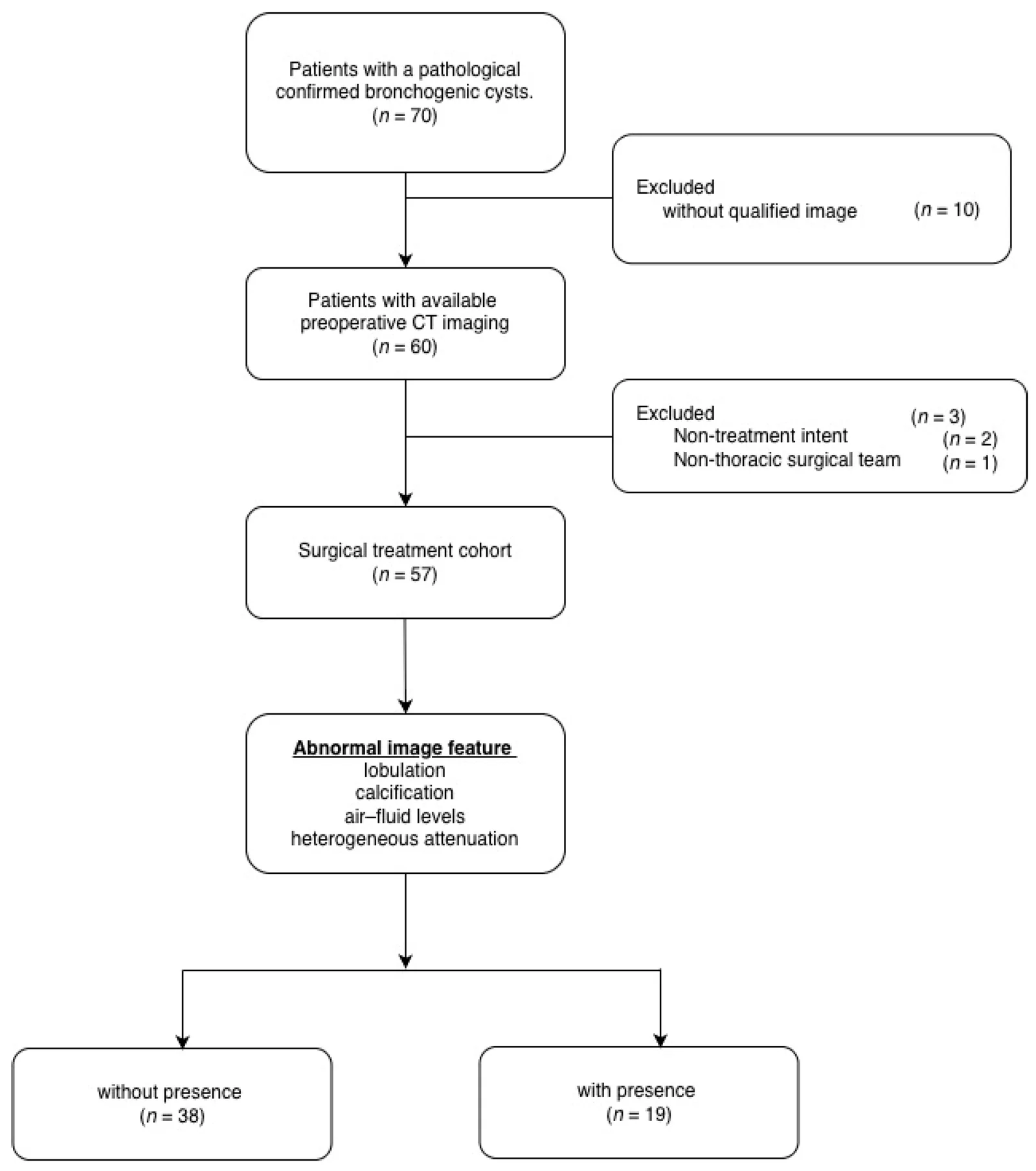
Flowchart of patient selection and computed tomography (CT) imaging features.

Patients were excluded if the surgery was not performed with curative intent or if it was conducted by a non-thoracic surgical team (Figure 1). The inclusion criterion was having at least one CT scan; patients whose CT images lacked Hounsfield-unit data were excluded. Collected data included demographic details (such as age and date of diagnosis), clinical symptoms, medical history, indications and timing of surgery, surgical methods, perioperative complications, and perioperative results. Symptom status was determined from preoperative outpatient clinic and admission notes using the definitions reported by Fievet et al. [12]. Patients were classified as symptomatic when at least one qualifying symptom was explicitly documented before surgery and as asymptomatic when no qualifying symptom was documented in either source. No imputation of symptom status was performed.

The study was approved by the Institutional Review Board of National Taiwan University Hospital (approval number 202301163RINB; Date: 10 March 2023). The requirement for informed consent was waived owing to the retrospective nature of the study.

### 2.2. Image Analysis

Preoperative imaging assessment of BCs relied primarily on CT scans, and magnetic resonance imaging was not routinely required. For each participant who underwent a chest CT scan (General Electric, Boston, MA, USA/Siemens Healthineers, Erlangen, Germany) at the National Taiwan University Hospital and National Taiwan University Cancer Center, the lung and mediastinal image series were reconstructed with a slice thickness of 1.00–1.25 mm. Preoperative chest CT examinations were retrospectively reviewed. For all patients, however, the four prespecified imaging features were assessed exclusively on unenhanced images. In patients who received intravenous contrast, the precontrast series was used for feature classification and Hounsfield-unit measurement; postcontrast images were not used in the study analysis.

Imaging analysis was overseen by Hsao-Hsun Hsu, a senior thoracic surgeon with more than 30 years of experience. Pei-Hsing Chen (11 years) and Kai-Man U (5 years) interpreted the scans and delineated the lesions of interest. All preoperative CT examinations were subsequently independently re-evaluated by Yu-Sen Huang, a radiologist more than 15 years of experience in thoracic imaging. The radiologist was blinded to the patients’ clinical symptoms, surgical procedures, intraoperative findings, postoperative outcomes, and the original imaging classifications assigned by the surgical team. Discordant interpretations were jointly reviewed by the radiologist and surgical readers and resolved by consensus. The final radiologist-confirmed consensus classifications were used in all outcome analyses. Imaging assessments were performed using features evaluable on both contrast-enhanced and non-contrast CT examinations to facilitate potential application to non-contrast low-dose CT screening. Hounsfield-unit measurements were obtained from the intracystic content using a consistent region-of-interest approach, avoiding the cyst wall, calcifications, and adjacent vascular structures.

**Figure 2:**
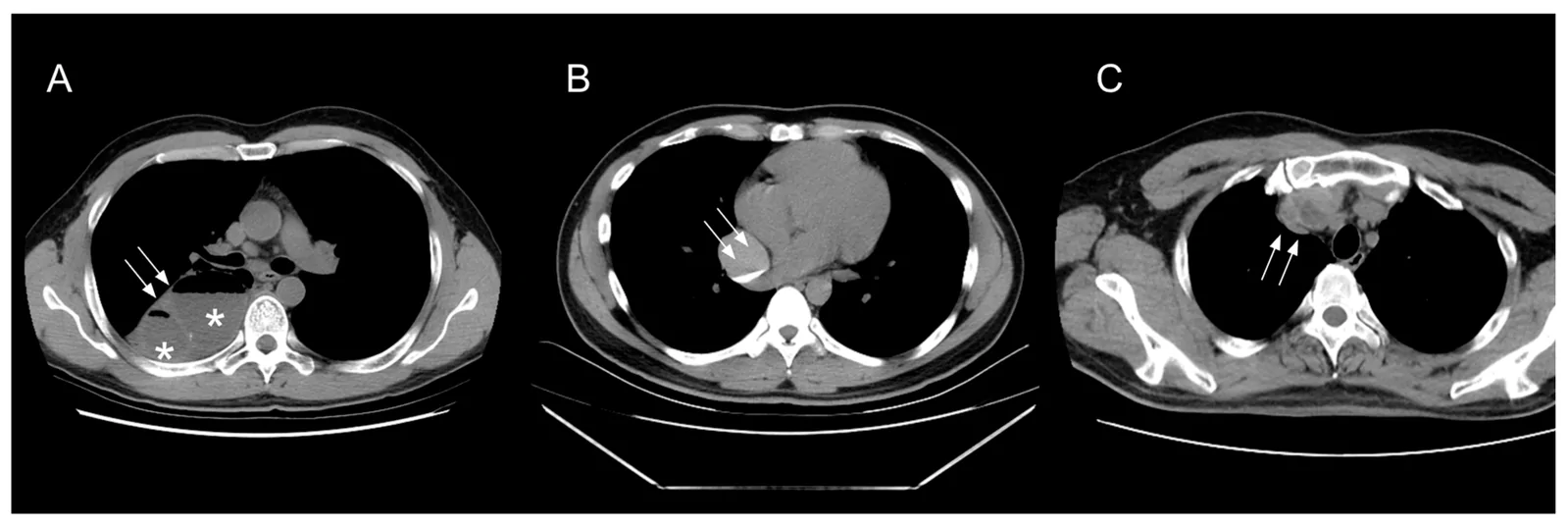
Representative preoperative computed tomography (CT) features of bronchogenic cysts. (**A**) Lobulated cyst margin (paired arrows) with an internal air–fluid level (asterisks) and dependent fluid layering. (**B**) Rim calcification (paired arrows). (**C**) Heterogeneous attenuation manifesting as areas with higher and lower densities (paired arrows) throughout the lesion.

The cyst morphology, attenuation, homogeneity of the cyst contents, calcification, and cyst-wall characteristics were assessed using the criteria described by McAdams et al. [13]. The composite classification of any abnormal CT imaging feature was defined as the presence of at least one of the following four prespecified features: lobulation, calcification, an air–fluid level, or heterogeneous attenuation (Figure 2). These features were not mutually exclusive. The composite classification was used as the principal imaging variable, whereas analyses of individual imaging features were considered exploratory. Lobulation was defined as the presence of more than one compartment within the lesion on the scan. Heterogeneous attenuation was defined as visually mixed densities within a cyst on soft-tissue CT windows (width 350, level 50). Mean attenuation of <20 Hounsfield units (HU) denoted fluid-like content, whereas that of >20 HU indicated soft-tissue–like content.

Cyst size was defined as the maximum diameter measured along the axial plane on CT scans. The location of the cyst was recorded as anterior mediastinal if it was between the anterior border of the heart or great vessels and sternum; middle mediastinal, if it was at the paratracheal region, close to the carina, or at the paraesophageal region; and posterior mediastinal, if it was at the paraspinal region [14]. Bronchogenic cyst location was additionally categorized according to the Maier classification as type I (paratracheal), type II (carinal or subcarinal), type III (hilar), type IV (paraesophageal), or type V (miscellaneous).

### 2.3. Preoperative Evaluation and Surgical Detail

Physical examination, clinical history, electrocardiography, standard laboratory tests, and a standard chest radiograph were obtained at admission. The CT of the thorax was repeated after admission if the most recent CT was older than 6 months. Patients’ comorbidities were categorized based on the Charlson Comorbidity Index.

Patients underwent general anesthesia and were intubated with either a single-lumen endotracheal tube with a blocker or a double-lumen endotracheal tube for single-lung ventilation. Patients were routinely positioned in the lateral decubitus position; a semi-lateral decubitus position was selected for anterior mediastinal cysts at the surgeon’s discretion. Thoracoscopic visualization was achieved using a 30° rigid telescope (Karl Storz SE & Co. KG, Tuttlingen, Germany, or Olympus Corporation, Tokyo, Japan). All lesions were treated to achieve margin-free resection.

### 2.4. Perioperative Outcomes and Follow-Up

The prespecified primary perioperative outcomes were peri-cystic adhesion, operative time, estimated intraoperative blood loss, and ICU length of stay. Secondary outcomes included chest-tube duration, postoperative hospital length of stay, major intraoperative complications, 30-day postoperative complications, and recurrence. Peri-cystic adhesions are defined as the presence of dense fibrotic attachments requiring sharp dissection, recorded intraoperatively. Chest tubes were placed routinely after the surgery and removed when the daily fluid drainage was <150 cc/day. The secondary endpoints comprised (i) Major intraoperative complications were defined as ClassIntra v1.0 grade ≥III events, including lung, esophageal, diaphragmatic, or phrenic-nerve injuries [15] and (ii) other postoperative complications. ClassIntra v1.0 grades intraoperative adverse events from grade 0, representing no deviation from the ideal intraoperative course, to grade V, representing intraoperative death. In this study, ClassIntra grade ≥III was considered a major intraoperative complication. The Clavien–Dindo classification grades postoperative complications from grade I to grade V according to the required treatment and clinical severity; grade III indicates a complication requiring surgical, endoscopic, or radiological intervention.

Postoperative complications occurring during the index hospitalization or within 30 days after discharge were recorded and graded with the Clavien–Dindo classification system [16]. Recurrence was determined radiographically using serial postoperative CT examinations and was defined as a new cystic lesion at or adjacent to the resection site or interval enlargement of documented residual cystic tissue. Residual tissue visible on the initial postoperative CT after incomplete resection was classified as residual disease and was not considered recurrence unless subsequent CT demonstrated interval enlargement. Repeat surgery or histopathological confirmation was not required. Time to recurrence was calculated from surgery to the first CT demonstrating recurrence or progression, whereas patients without recurrence, including those with short follow-up, were censored at their last CT examination. Because this was a retrospective study of a benign disease, surveillance was not performed according to a prespecified protocol; follow-up imaging was generally initiated 6–12 months after surgery, with longer intervals thereafter according to clinical practice.

### 2.5. Statistical Analysis

Statistical analysis was performed using SPSS 27.0 (IBM, Armonk, NY, USA). Continuous variables are summarized as mean ± standard deviation. Group comparisons for continuous variables were conducted using the 2-tailed independent t-test. Categorical variables are presented as numbers and percentages, with statistical comparisons performed using the Chi-square test or Fisher’s exact test if the expected value is less than five. A *p* value of <0.05 was considered statistically significant in all analyses. Feature-specific analyses are presented in Supplementary Table S2. Recurrence-free survival was estimated using the Kaplan–Meier method and compared between groups using the log-rank test. Median follow-up duration was estimated using the reverse Kaplan–Meier method, and follow-up distributions were compared using a log-rank test applied to the reverse Kaplan–Meier curves. These analyses were considered exploratory because of the small number of recurrence events. Multivariate analyses were performed using logistic regression for categorical variables and linear regression for continuous variables. To ensure the stability and interpretability of our multivariable models, collinearity among candidate independent variables was evaluated using the variance inflation factor. ChatGPT (GPT-5.6, OpenAI, San Francisco, CA, USA; https://chatgpt.com; accessed 8 September 2026) was used solely for English-language editing. All AI-assisted text was reviewed and approved by the authors.

## 3. Results

### 3.1. Patient Characteristics

Among 70 pathologically confirmed bronchogenic cyst cases, 9 lacked preoperative CT imaging, specimens in 2 cases were obtained during open-heart surgery, 1 patient was managed by the pediatric surgical team, and 1 had a lesion measuring <6 mm on CT. Consequently, 57 patients (36 women and 21 men) met the inclusion criteria for the final analysis (Figure 1). Most patients presented with Eastern Cooperative Oncology Group performance status scores of 0 to 1 (55, 96.5%), with cysts located in the posterior mediastinum (36, 63.2%), and had current or previous smoking history (11, 19.3%; Table 1). Although 52 patients had an additional contrast-enhanced series available, all imaging-feature classifications and Hounsfield-unit measurements were based on unenhanced images. Abnormal imaging was also associated with higher thoracotomy rates (15.8% vs. 0%, *p* = 0.002), more concomitant lung resections, and a greater prevalence of intrapulmonary lesions (42.1% vs. 2.6%, *p* < 0.001) (Table 1).

### 3.2. Clinical Characteristics of Patients With or Without Abnormal Imaging Features

Of the 57 patients, 52 (91.2%) received contrast-enhanced preoperative CT, 5 underwent non-contrast CT only, and 19 (33.3%) displayed abnormal imaging features. Patients with abnormal imaging were more often male (57.9% vs. 26.3%, *p* = 0.040), symptomatic (73.7% vs. 31.6%, *p* = 0.004), and received preoperative drainage/biopsy more frequently (31.6% vs. 2.6%, *p* = 0.004). Their cysts were larger (mean 5.09 ± 2.58 cm vs. 3.17 ± 1.49 cm, *p* = 0.006) and most often displayed lobulation (63.2%), heterogeneous attenuation (57.9%), calcification (36.8%), or an air–fluid level (21.1%) (Table 1).

### 3.3. Perioperative Outcomes

Patients with abnormal imaging features showed a higher rate of peri-cystic adhesion (68.3% vs. 34.2%; *p* = 0.015), longer mean operating times (127.8 ± 59.8 vs. 86.8 ± 34.5 min; *p* = 0.011), greater intra-operative blood loss (100.0 ± 150.0 vs. 3.9 ± 24.3 mL; *p* = 0.012), and a longer postoperative ICU stay (0.84 ± 1.21 vs. 0.05 ± 0.23 days; *p* = 0.011) compared with those with normal imaging (Table 2). Drainage-tube duration and total postoperative length of stay did not differ significantly between the two groups, and no 30-day mortality or readmissions occurred in either cohort. Five radiographic recurrence events were identified: three among patients with abnormal imaging features and two among those without abnormal imaging features (15.8% vs. 5.3%, respectively; two-sided Fisher’s exact *p* = 0.321). Median recurrence-free survival was not reached in either group, and recurrence-free survival did not differ significantly between groups (log-rank *p* = 0.294). The reverse Kaplan–Meier estimates of median follow-up were 72.8 and 60.4 months, respectively, with no statistically significant difference in follow-up distributions (*p* = 0.415; Supplementary Table S5). In the exploratory multivariable models presented in Table 3, the composite abnormal CT imaging classification remained associated with longer operative time, greater intraoperative blood loss, and longer ICU stay (*p* = 0.027, 0.039, and 0.023, respectively) after adjustment for age, sex, smoking status, ECOG performance status, Charlson Comorbidity Index, cyst size, and preoperative drainage or biopsy. Because symptom status was not included in these models, these findings should not be interpreted as demonstrating an association independent of symptoms or incremental predictive value beyond symptom status. In the joint multivariable model for peri-cystic adhesion, symptom status remained significantly associated with adhesion (adjusted OR, 5.564; 95% CI, 1.225–25.276; *p* = 0.026), whereas the composite abnormal CT imaging classification was not independently associated with adhesion after adjustment for symptom status (adjusted OR, 1.403; 95% CI, 0.283–6.958; *p* = 0.678; Table S1).

Table S2 shows that specific CT findings portend differing perioperative courses: lobulation (*n* = 12) was strongly associated with higher adhesion rates (92% vs. 33%, *p* < 0.001), longer operative time (147 ± 66 vs. 88 ± 33 min, *p* = 0.011), greater blood loss (158 ± 164 vs. 3 ± 22 mL, *p* = 0.007), prolonged ICU stay (1.2 ± 1.4 vs. 0.1 ± 0.3 days, *p* = 0.022), longer drainage (4.9 ± 3.8 vs. 2.2 ± 3.5 days, *p* = 0.033), and extended hospitalization (6.0 ± 3.6 vs. 3.6 ± 3.5 days, *p* = 0.046); calcification (*n* = 7) showed non-significant trends toward poorer outcomes; the presence of an air–fluid level (*n* = 4) predicted 100% adhesion and markedly longer surgery (178 ± 36 vs. 95 ± 44 min, *p* < 0.001), as well as significantly longer ICU stays, drainage duration, and hospital stays (all *p* < 0.05); and heterogeneous attenuation (*n* = 11) was linked to longer surgery (145 ± 64 vs. 90 ± 37 min, *p* = 0.017), greater blood loss (150 ± 180 vs. 9 ± 30 mL, *p* = 0.027), and extended ICU and hospital stays (*p* = 0.010 and *p* = 0.023, respectively), although adhesion and drainage durations were unaffected. Patients without lobulation or air–fluid levels demonstrated significantly better perioperative outcomes. Patients with calcification showed a trend toward poorer perioperative results, although the results were not statistically significant (Table S2).

Intra-operative complications were documented in eight cases: esophageal (*n* = 3), lung (*n* = 2), diaphragm (*n* = 1), and suspected phrenic-nerve (*n* = 1) injuries. Post-operatively, only one patient experienced a Clavien–Dindo grade ≥3 event—esophageal perforation with empyema—necessitating esophageal stent placement and pleural decortication on postoperative day 14; the stent was removed 1.5 months later (Table S3; Table S4).

Table 4 summarizes the perioperative outcomes stratified by symptom status. Of the 31 asymptomatic patients, only 8 (25.8%) had adhesions around the cyst, compared with 18 of the 26 symptomatic patients (69.2%; *p* = 0.001). The mean operative time was significantly shorter in the asymptomatic group (81.1 ± 31.2 min vs. 123.5 ± 54.8 min; *p* = 0.011) and mean intraoperative blood loss was markedly lower (4.8 ± 26.9 vs. 73.1 ± 135.1 mL; *p* = 0.017). No asymptomatic patient required intensive-care admission, whereas symptomatic patients spent an average of 0.7 ± 1.1 days in the ICU (*p* = 0.003). Drainage-tube duration and total postoperative hospital stay were also significantly shorter in the asymptomatic cohort (1.5 ± 0.9 vs. 4.0 ± 4.8 days, *p* = 0.013; and 3.0 ± 1.4 days vs. 5.5 ± 4.9 days, *p* = 0.018, respectively). Cyst recurrence rates did not differ between groups (9.7% vs. 7.7%; *p* = 0.585). In the joint multivariable logistic regression model for peri-cystic adhesion, symptom status remained significantly associated with adhesion (adjusted OR, 5.564; 95% CI, 1.225–25.276; *p* = 0.026), whereas abnormal CT imaging features were not independently associated with adhesion after adjustment for symptom status (adjusted OR, 1.403; 95% CI, 0.283–6.958; *p* = 0.678).

## 4. Discussion

This observational cohort study found that the presence of lobulation, calcification, air–fluid levels, or heterogeneous attenuation was associated with less favorable perioperative outcomes, including higher rates of peri-cystic adhesion, longer operative times, increased blood loss, and extended ICU stays. In factor-specific analyses, each CT feature, especially lobulation and an air–fluid level, showed a strong trend toward less favorable perioperative outcomes. These findings suggest that CT features may help identify patients at increased perioperative risk; however, they do not establish a causal relationship.

Current surgical consensus holds that BCs should be resected when symptomatic or complicated; however, the management of asymptomatic, uncomplicated cysts remains contentious. Reported rates of symptomatic presentation range from 29–94% [17,18]. Kirmani et al. reviewed 23 best-evidence reports encompassing 683 adults with BCs, of whom 74 were initially managed conservatively or observed. Nearly half (45%) of these patients developed symptoms and required surgery [5]. Radiographic surveillance of BCs often extends over decades, placing a prolonged and repeated imaging burden on patients. However, there are no evidence-based guidelines defining optimal follow-up intervals. In pediatric cases, lifelong CT monitoring raises concerns regarding cumulative radiation exposure and the psychological impact on children and their families. Notably, spontaneous resolution of BCs has not been documented, underscoring the need for clear monitoring strategies [5]. These data indicate that a substantial proportion of ostensibly asymptomatic cysts will eventually necessitate resection. Some authors advocate excising all BCs to prevent infectious complications; however, postoperative morbidity can be as high as 27% [12]. Moreover, previous series report that 25–37% of BCs progress to severe complications if left untreated [19,20].

Fievet et al. reported that adult patients were markedly more likely to be symptomatic than pediatric patients (58.5% vs. 41%, *p* < 0.05). Symptomatic adults underwent more open resections, experienced higher conversion rates from thoracoscopy to thoracotomy, and required additional intraoperative procedures than their asymptomatic counterparts. Pathological analysis revealed a greater incidence of inflammatory reactions in symptomatic adults (65.5% vs. 40%, *p* = 0.006), likely accounting for the increased surgical complexity. Symptomatic patients also had their postoperative stay extended by almost 2 days (*p* < 0.001). However, their overall complication rates did not differ meaningfully from those in asymptomatic patients. We compared the symptomatic and asymptomatic patients in our cohort (Table 4). Symptomatic status was associated with significantly worse perioperative outcomes, including higher rates of peri-cystic adhesion, longer operative times, increased blood loss, prolonged ICU stays, extended drainage duration, and longer postoperative hospitalization. However, it did not predict recurrence.

Symptomatic patients frequently report respiratory issues, especially after cyst enlargement or infection [21]. Fievet et al. [12] showed that symptomatic status is associated with perioperative outcomes and may serve as a surrogate for local inflammation; however, symptoms are inherently subjective and can vary between individuals, thereby reducing their reliability. Since 2006, Chang et al. have demonstrated that specific CT features correlate closely with clinical presentation and histopathologic findings. In their study, contrast-enhanced CT scans were categorized by cystic content into three groups (those exhibiting fluid attenuation only, those showing air–fluid levels, and those displaying heterogeneous soft-tissue attenuation), thereby linking imaging characteristics to patient symptoms and underlying pathology [22]. In this study, we assessed clinical and histopathologic correlations using the CT imaging features defined by McAdams and Cleveland [13,23,24]—namely, lobulation, calcification, air–fluid levels, and heterogeneous attenuation. Because these features may coexist and may represent overlapping manifestations of complicated cyst contents, inflammation, or other processes associated with operative difficulty, we retained the original four prespecified features and analyzed the presence of any of these features primarily as a composite abnormal-imaging classification rather than emphasizing any single feature. This approach also reduces the risk of overinterpreting individual features with small subgroup sizes or greater reader-dependent variability. Accordingly, the composite classification was considered the principal imaging variable, whereas the individual-feature analyses were exploratory. Wall enhancement is a recognized parameter; however, its accurate assessment requires contrast-enhanced imaging and is not feasible on non-contrast CT screening scans. Therefore, we excluded it to maintain applicability in the CT screening era.

Notably, several key preoperative and intraoperative factors must be considered when planning BC resection. Cyst size has repeatedly been shown to influence the choice of surgical approach, with lesions < 5 cm reliably amenable to VATS and larger cysts more often requiring thoracotomy [25]. Although size shows correlations with some perioperative outcomes, its predictive value warrants further validation (Table 3; Table S1). Dense adhesions to vital structures, such as the airway, major vessels, or pleura, further increase the likelihood of conversion from VATS to open surgery and may necessitate subtotal resection to avoid injury [26]. Symptomatic presentation, often reflecting cyst enlargement or secondary infection, correlates with higher rates of perioperative complications and suggests that earlier intervention may improve outcomes [27]. The anatomical location also plays a role, as mediastinal cysts are generally more accessible through VATS, whereas intrapulmonary or cervical lesions may pose greater technical challenges and distinct complication profiles [28]. The current strategy of early resection for asymptomatic or small cysts to minimize adhesion formation, preserve lung parenchyma, and prevent frequent radiation exposure is consistent with long-standing recommendations in the literature [29]. These factors underscore the importance of individualized operative planning to optimize safety and efficacy in bronchogenic cyst management. VATS is generally preferred for resectable mediastinal cysts because of its minimally invasive nature and favorable perioperative recovery, and its feasibility has been supported by contemporary series of videothoracoscopic mediastinal surgery [30]. However, abnormal CT features should not be considered absolute indications for thoracotomy. Rather, these features may identify cases with greater anticipated technical complexity and support involvement of an experienced thoracic surgeon, preparation for possible conversion, and individualized selection between VATS and an open approach.

To our knowledge, this is the largest study to examine associations between CT imaging features and perioperative outcomes in bronchogenic cyst resection. These findings may support preoperative risk assessment beyond symptom status alone [12]. CT features may also be incorporated into future risk models, including quantitative or artificial intelligence-assisted approaches designed to reduce reader-dependent variability and improve reproducibility across radiologists and institutions, although prospective development and external validation are required.

However, this study has some limitations. The sample size was modest and obtained from a single center. Furthermore, the same imaging criteria were applied across examinations. All 57 patients had unenhanced CT images available; 52 also had contrast-enhanced series, whereas five had unenhanced images only. Because all four prespecified features and Hounsfield-unit measurements were assessed on unenhanced images, intravenous contrast did not directly influence these study measurements. Cyst-wall enhancement was not evaluated. However, differences in scanners, acquisition parameters, and CT protocols during the study period may have introduced residual variability between patients. We deliberately excluded contrast-dependent features, such as wall enhancement, to preserve applicability to non-contrast LDCT screening; nevertheless, future validation using a standardized non-contrast LDCT protocol will be important to confirm the applicability of these findings in the lung cancer screening setting. In addition, the retrospective design and the absence of a pathological evaluation of regional fibrosis may undermine the validity of the study findings. Retrospective symptom assessment may have resulted in under-ascertainment and temporal heterogeneity due to changes in physicians and documentation practices over the study period. Intraoperative outcomes, including peri-cystic adhesion, were also extracted from medical records and may therefore be subject to information bias. Although the binary composite was selected to preserve statistical stability, this dichotomization may obscure heterogeneity among imaging phenotypes. Larger studies are required to determine whether a weighted or count-based imaging score provides additional prognostic value. The nearly 20-year inclusion period encompassed changes in surgical and anesthetic practice, including accumulation of surgeon experience, adoption of multiportal and uniportal VATS, and changes in postoperative and ICU admission practices. No formal time-trend or calendar-period-adjusted analysis was performed because of the modest cohort size and limited number of major events. Therefore, residual confounding related to surgical era and learning-curve effects cannot be excluded. Although all CT examinations were independently reviewed by a radiologist and discordant interpretations were resolved by consensus, reader-dependent variability remained for individual imaging features, particularly heterogeneous attenuation. Moreover, assessment by a single radiologist cannot fully establish reproducibility across radiologists with different levels of experience or across institutions. Therefore, although the outcome analyses were based on the final radiologist-confirmed consensus classifications, multicenter studies involving multiple independent readers are required to confirm the generalizability of these findings. Finally, although we assessed collinearity using the variance inflation factor, we did not include other covariates, such as specific cyst characteristics or their interactions, in this analysis.

In conclusion, prespecified imaging features assessed on unenhanced CT images were associated with less favorable perioperative outcomes in patients undergoing bronchogenic cyst resection. Incorporating these imaging features into preoperative assessment may support risk stratification and inform surgical planning in the lung cancer CT screening era. Future multicenter prospective studies should validate these cutoffs and evaluate their integration into clinical decision algorithms.

## Figures and Tables

**Table 1:** Patient demographics.

		All (*n* = 57)	Abnormal Imaging Features		*p* Value
			Without (*n* = 38)	With (*n* = 19)	
Male sex		21 (36.8)	10 (26.3)	11 (57.9)	0.040
Age (years)		43.3 ± 15.3	43.6 ± 15.7	42.6 ± 15.0	0.821
Smoker		11 (19.3)	6 (15.8)	5 (26.3)	0.478
ECOG	0	42 (73.7)	28 (73.7)	14 (73.7)	>0.999
	1	13 (22.8)	9 (23.7)	4 (21.1)	
	2	2 (3.5)	1 (2.6)	1 (5.3)	
Charlson Comorbidity Index	0	30 (52.6)	20 (52.6)	10 (52.7)	0.947
	1	12 (21.1)	7 (18.4)	5 (26.3)	
	2	7 (12.3)	5 (13.2)	2 (10.5)	
	3	7 (12.3)	5 (13.2)	2 (10.5)	
	4	1 (1.8)	1 (2.6)	0	
Clinical symptoms (%)		26 (45.6)	12 (31.6)	14 (73.7)	0.004
Preoperative drainage or biopsy (%)		7 (12.3)	1 (2.6)	6 (31.6)	0.004
Cyst location	Anterior mediastinum (%)	11 (19.3)	9 (23.7)	2 (10.5)	0.073
	Middle mediastinum (%)	6 (10.5)	3 (7.9)	3 (15.8)	
	Posterior mediastinum (%)	36 (63.2)	25 (65.8)	11 (57.9)	
	Intrapulmonary (%)	4 (7.0)	1 (2.63)	3 (15.8)	
Maier classification	Type I (paratracheal) (%)	7 (12.3)	4 (10.5)	3 (15.8)	0.073
	Type II (subcarinal) (%)	10 (17.5)	7 (18.4)	3 (15.8)	
	Type III (hilar) (%)	9 (15.8)	4 (10.5)	3 (15.8)	
	Type IV (paraesophageal) (%)	9 (15.8)	6 (15.8)	3 (15.8)	
	Type V (Others) (%)	22 (38.6)	17 (44.7)	5 (26.3)	
Cyst diameter (cm)		3.81 ± 2.11	3.17 ± 1.49	5.09 ± 2.58	0.006
HU of cyst content		34.7 ± 28.8	32.1 ± 31.3	40.0 ± 22.9	0.330
Additional contrast-enhanced series available, *n* (%)		52 (91.2)	34 (89.2)	18 (94.7)	0.655
Surgical approaches	Thoracotomy (%)	3 (5.3)	0	3 (15.8)	0.002
	Multiportal VATS (%)	38 (66.7)	23 (60.5)	15 (79.0)	
	Uniportal VATS (%)	16 (28.1)	15 (39.5)	1 (5.3)	
Surgical procedures	Cyst resection only (%)	48 (84.2)	37 (97.4)	11 (57.9)	<0.001
	Combined with lung resection (%)	9 (15.8)	1 (2.6)	8 (42.1)	

Abbreviations: ECOG, Eastern Cooperative Oncology Group; HU, Hounsfield units; VATS, video-assisted thoracoscopic surgery.

**Table 2:** Prespecified primary perioperative and selected secondary outcomes according to abnormal imaging features.

	Abnormal Imaging Features		*p* Value
	Without (*n* = 38)	With (*n* = 19)	
Peri-cystic adhesions (%)	13 (34.2)	13 (68.3)	0.015
Operation time (minutes)	86.8 ± 34.5	127.8 ± 59.8	0.011
Blood loss (mL)	3.9 ± 24.3	100.0 ± 150.0	0.012
ICU stays (days)	0.05 ± 0.23	0.84 ± 1.21	0.011
Chest-tube duration (days)	2.27 ± 3.85	3.82 ± 3.41	0.173
Postoperative hospital length of stay (days)	3.66 ± 3.83	5.11 ± 3.14	0.160
Cyst recurrence (%)	2 (5.3)	3 (15.8)	0.321

Abbreviation: ICU, intensive care unit.

**Table 3:** Multivariate analysis of perioperative outcomes.

	Operation Time (mins)			Blood Loss (cc)			ICU Stay (days)		
	Coefficient (B)	*p* Value	VIF	Coefficient (B)	*p* Value	VIF	Coefficient (B)	*p* Value	VIF
Age	0.860 (−0.403–2.123)	0.177	2.586	0.519 (−1.889–2.928)	0.667	2.586	−0.001 (−0.020–0.018)	0.934	2.586
Sex (male)	−17.525 (−51.006–15.956)	0.298	1.828	−47.118 (−110.966–16.730)	0.144	1.828	−0.522 (−1.021–−0.024))	0.040	1.828
Smoker	8.615 (−33.812–51.041)	0.685	1.964	34.782 (−46.125–115.688)	0.392	1.964	0.295 (−0.337–0.926)	0.353	1.964
ECOG	−12.258 (−43.653–19.138)	0.436	1.930	7.242 (−52.630–67.113)	0.809	1.930	−0.259 (−0.726–0.209)	0.271	1.930
CCI	−4.230 (−21.688–13.228)	0.628	2.749	−9.285 (−42.577–24.008)	0.578	2.749	0.094 (−0.166–0.354)	0.469	2.749
Abnormal CT imaging feature	34.322 (3.993–64.651)	0.027	1.432	61.061 (3.223–118.898)	0.039	1.432	0.527 (0.076–0.979)	0.023	1.432
Cyst size	6.317 (−1.255–13.889)	0.100	1.756	16.579 (2.139–31.018)	0.025	1.756	0.133 (0.020–0.246)	0.022	1.756
Preoperative drainage	−1.160 (−45.989–43.670)	0.959	1.517	45.573 (−39.916–131.063)	0.289	1.517	0.556 (−0.112–1.223)	0.101	1.517

Abbreviations: CCI, Charlson comorbidity index; CT, computed tomography; ECOG, Eastern Cooperative Oncology Group; ICU, intensive care unit; VIF, variance inflation factor.

**Table 4:** Perioperative results in patients with different symptoms.

	Symptom		*p* Value
	Without (*n* = 31)	With (*n* = 26)	
Peri-cystic adhesions (%)	8 (25.8)	18 (69.2)	0.001
Operation time (minutes)	81.1 ± 31.2	123.5 ± 54.8	0.011
Blood loss (mL)	4.8 ± 26.9	73.1 ± 135.1	0.017
ICU stays (days)	0.0 ± 0.0	0.7 ± 1.1	0.003
Drainage tube duration (days)	1.5 ± 0.9	4.0 ± 4.8	0.013
Postoperative stays(days)	3.0 ± 1.4	5.5 ± 4.9	0.018
Cyst recurrence (%)	3 (9.7)	2 (7.7)	>0.999

Abbreviation: ICU, intensive care unit.

## Data Availability

Data generated or analyzed during the study are available from the corresponding author by request.

## Supplementary Materials

The following supporting information can be downloaded at: https://www.mdpi.com/article/10.3390/diagnostics16183000/s1, Table S1: Multivariable analysis of the prespecified primary perioperative outcome of peri-cystic adhesion. Table S2: Prespecified primary perioperative and selected secondary outcomes according to individual CT imaging features; Table S3: Secondary outcome: intraoperative complications according to the presence of abnormal CT imaging features; Table S4: Secondary outcome: intraoperative complications according to individual CT imaging features; Table S5: Secondary outcome: recurrence and time-to-event analyses according to abnormal CT imaging features.

## Abbreviations

The following abbreviations are used in this manuscript:

BC	Bronchogenic cyst
CCI	Charlson comorbidity index
CT	Computed tomography
ECOG	Eastern Cooperative Oncology Group
HU	Hounsfield unit
ICU	Intensive care unit
VATS	Video-assisted thoracoscopic surgery
VIF	Variance inflation factor
