# Supplementary material for: Association Between Unenhanced CT Features and Perioperative Outcomes in Bronchogenic Cyst Resection
Source: Diagnostics (Basel). 2026 Sep 16;16(18):3000. doi: 10.3390/diagnostics16183000 (PMC13606000; doi:10.3390/diagnostics16183000)
Supplement: Supplementary file 1 [file diagnostics-16-03000-s001.zip › diagnostics-4458489-supplementary.pdf]

Supplementary Table S1. *Multivariable analysis of the prespecified primary perioperative outcome of peri-cystic adhesion.*

Characteristic	OR	95%CI	*p*
**Model 1: with clinical data and CT imaging features**			
Age	0.994	0.942–1.049	0.829
Sex (male)	3.098	0.630–15.243	0.164
Smoker	0.153	0.019–1.226	0.077
ECOG	1.622	0.377–6.989	0.516
CCI	0.775	0.344–1.746	0.539
Abnormal CT imaging features	3.811	1.056–13.753	0.041
**Model 2: with all related clinical factors, CT imaging features, and symptoms**			
Age	1.011	0.943–1.084	0.751
Sex (male)	4.076	0.574–28.955	0.160
Smoker	0.081	0.006–1.119	0.061
ECOG	4.195	0.704–25.017	0.115
CCI	0.638	0.236–1.726	0.376
Symptom	5.564	1.225–25.276	0.026
Abnormal CT imaging features	1.403	0.283–6.958	0.678
Cyst size	1.609	0.958–2.703	0.072
Preoperative drainage	0.325	0.028–3.825	0.372

CCI, Charlson comorbidity index; CI, confidence interval; ECOG, Eastern Cooperative Oncology Group; OR, Odds ratio.

Supplementary Table S2. *Prespecified primary perioperative and selected secondary outcomes according to individual CT imaging features.*

	**Lobulation**			**Calcification**			**Air-fluid level**			**Heterogeneous attenuation**		
	With (*n*=12)	Without (*n*=45)	*p* value	With (*n*=7)	Without (*n*=50)	*p* value	With (*n*=4)	Without (*n*=53)	*p* value	With (*n*=11)	Without (*n*=46)	*p* value
Peri-cystic adhesions (%)	11 (91.7)	15 (33.3)	<0.001	4 (57.1)	22 (44.0)	0.399	4 (100)	22 (41.5)	0.038	7 (63.6)	19 (41.3)	0.159
Operation time (minutes)	146.7±66.3	88.1±33.4	0.011	110.4±46.9	99.1±48.6	0.563	178.0±36.3	94.6±43.8	<0.001	144.9±63.5	89.8±37.3	0.017
Blood loss (ml)	158.3±163.5	3.3±22.4	0.007	78.6±186.8	30.0±80.8	0.521	275.0±170.8	17.9±63.6	0.056	150.0±180.3	8.7±30.4	0.027
ICU stays (days)	1.17±1.40	0.09±0.29	0.022	0.43±0.79	0.30±0.81	0.696	2.25±1.26	0.17±0.55	0.044	1.36±1.36	0.07±0.25	0.010
Drainage tube duration (days)	4.91±3.78	2.19±3.54	0.033	4.17±5.04	2.63±3.55	0.354	7.25±4.72	2.42±3.42	0.012	4.55±4.01	2.31±3.54	0.082
Postoperative hospital stays (days)	6.00±3.64	3.64±3.53	0.046	5.43±4.35	3.96±3.56	0.323	8.75±4.19	3.79±3.41	0.008	6.36±3.53	3.61±3.51	0.023

ICU, intensive care unit.

Supplementary Table S3. *Secondary outcome: intraoperative complications according to the presence of abnormal CT imaging features.*

	**All population (*n*=57)**	**Abnormal imaging features**		***p* value**
		Without (*n*=38)	With (*n*=19)	
Intraoperative complications (Total, %)	8 (14.0)	5 (13.2)	3 (15.8)	>0.999
Lung injury (%)	3 (5.2)	2 (5.3)	1 (5.3)	
Esophageal injury (%)	3 (5.2)	2 (5.3)	1 (5.3)	
Diaphragm injury (%)	1 (1.8)	0	1 (5.3)	
Phrenic nerve injury (%)	1 (1.8)	1 (2.6)	0	

Supplementary Table S4. *Secondary outcome: intraoperative complications according to individual CT imaging features.*

	Lobulation			Calcification			Air-fluid level			Heterogeneous attenuation		
	With (n=12)	Without (n=45)	p value	With (n=7)	Without (n=50)	p value	With (n=4)	Without (n=53)	p value	With (n=11)	Without (n=46)	p value
Intraoperative complications (%)	1 (8.3)	7 (15.6)	>0.999	1 (14.3)	7 (14)	>0.999	0	8 (15.1)	>0.999	2 (18.2)	6 (13.0)	0.644
Lung injury (%)	1 (8.3)	2 (4.4)		1 (14.3)	2 (4)		0	3 (5.7)		0	3 (6.5)	
Esophageal injury (%)	0	3 (6.7)		0	3 (6)		0	3 (5.7)		1 (9.1)	2 (4.3)	
Diaphragm injury (%)	0	1 (2.2)		0	1 (2)		0	1 (1.9)		1 (9.1)	0	
Phrenic nerve injury (%)	0	1 (2.2)		0	1 (2)		0	1 (1.9)		0	1 (2.2)	

Supplementary Table S5. *Secondary outcome: recurrence and time-to-event analyses according to abnormal CT imaging features.*

	**Abnormal imaging features**		***p* value**
	Without (*n*=38)	With (*n*=19)	
Recurrence events, *n* (%)	2 (5.3)	3 (15.8)	0.321
Median recurrence-free survival, months	NR	NR	0.294[a]
Median follow-up	60.4	72.8	0.415[b]

Abbreviations: CT, computed tomography; NR, not reached.

[a] Comparison of recurrence-free survival distributions using the log-rank test.

[b] Comparison of follow-up distributions using the reverse log-rank test. Neither comparison was statistically significant.
